# Treating COVID-19 with Medicinal Plants: Is It Even Conceivable? A Comprehensive Review

**DOI:** 10.3390/v16030320

**Published:** 2024-02-20

**Authors:** Hadi Al-Jamal, Sara Idriss, Rabih Roufayel, Ziad Abi Khattar, Ziad Fajloun, Jean-Marc Sabatier

**Affiliations:** 1 Faculty of Public Health 3, Lebanese University, Tripoli 1100, Lebanon; hadi.aljamal.1@outlook.com; 2 Laboratory of Applied Biotechnology (LBA3B), Azm Center for Research in Biotechnology and Its Applications, EDST, Lebanese University, Tripoli 1300, Lebanon; sarahidriss4@gmail.com; 3 College of Engineering and Technology, American University of the Middle East, Egaila 54200, Kuwait; rabih.roufayel@aum.edu.kw; 4 Faculty of Medicine and Medical Sciences, University of Balamand, Kalhat, Tripoli P.O. Box 100, Lebanon; ziad.abikhattar@balamand.edu.lb; 5 Department of Biology, Faculty of Sciences 3, Campus Michel Slayman Ras Maska, Lebanese University, Tripoli 1352, Lebanon; 6 INP, Inst Neurophysiopathol, Aix-Marseille Université, CNRS, 13385 Marseille, France

**Keywords:** SARS-CoV-2, COVID-19, virus, WHO, herbal medicine, bioactive compounds

## Abstract

In 2020, severe acute respiratory syndrome coronavirus-2 (SARS-CoV-2) challenged the world with a global outbreak that led to millions of deaths worldwide. Coronavirus disease 2019 (COVID-19) is the symptomatic manifestation of this virus, which can range from flu-like symptoms to utter clinical complications and even death. Since there was no clear medicine that could tackle this infection or lower its complications with minimal adverse effects on the patients’ health, the world health organization (WHO) developed awareness programs to lower the infection rate and limit the fast spread of this virus. Although vaccines have been developed as preventative tools, people still prefer going back to traditional herbal medicine, which provides remarkable health benefits that can either prevent the viral infection or limit the progression of severe symptoms through different mechanistic pathways with relatively insignificant side effects. This comprehensive review provides scientific evidence elucidating the effect of 10 different plants against SARS-CoV-2, paving the way for further studies to reconsider plant-based extracts, rich in bioactive compounds, into more advanced clinical assessments in order to identify their impact on patients suffering from COVID-19.

## 1. Introduction

**Figure 1:**
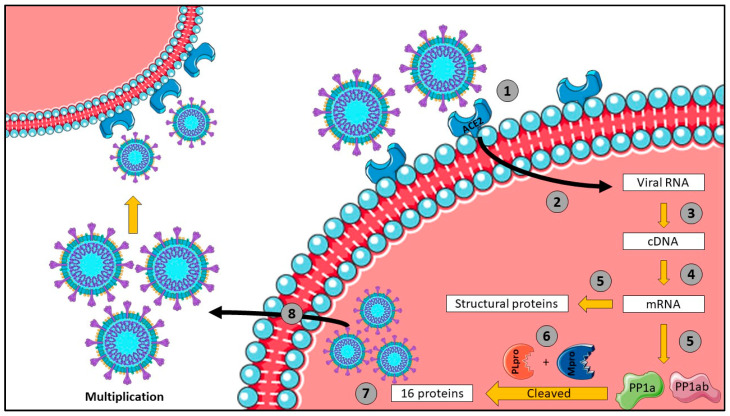
SARS-CoV-2 cellular infection scheme—1: spike protein adherence to angiotensin converting enzyme 2 (ACE2) membrane receptors; 2: viral entry; 3: reverse transcription of viral RNA into cDNA; 4: cDNA transcription into mRNA coding for proteins; 5: mRNA translation into PP1a, PP1ab, and structural proteins; 6: PP1a and PP1ab cleaved by papain-like (PL) protease and main protease enzymes to form 16 non-structural proteins; 7: viral assembly; 8: virus exits the cell to infect other hosts.

Coronavirus disease (COVID-19) is an acute respiratory syndrome that led to a global pandemic affecting our daily lives. Despite identifying the first clinical case in Wuhan, China in 2019, the virus continued to spread, leading to nearly 771 million cases, among which 7 million deaths occurred globally, based on the world health organization (WHO) [1]. This is due to the ability of the virus to spread quickly through direct (e.g., sneeze or cough droplets in the air) and indirect transmissions (e.g., nasal, and oral mucosal excretions on surfaces) [2]. SARS-CoV-2 is a single-stranded RNA virus belonging to the Coronavirinae family. It carries the necessary genes for the synthesis of 16 non-structural proteins by cleaving the originally two polypeptides pp1a and pp1ab. Actually, papain-like protease (PL_pro_) and chymotrypsin-like protease (3CL_pro_) enzymes are responsible for this cleavage [3]. In addition, their genome encodes for structural proteins, of which four are the most prominent: spike glycoproteins, membrane proteins, envelope proteins, and nucleocapsid proteins [4]. A simplified scheme of the SARS-CoV-2 infection is illustrated in Figure 1. The spike proteins (S-proteins) bind to a carboxypeptidase that degrades angiotensin II (Ang II). The receptor of SARS-CoV-2 is then angiotensin-converting enzyme type 2 (ACE2) receptors with their S1 subunits, while S2 subunits are responsible for the virus’s fusion and its internalization inside the host cells. Therefore, cleavage enzymes from the host, including transmembrane protease serine 2 (TMPRSS2), are needed to separate the two subunits for complete entry [5,6]. In fact, the high affinity of SARS-CoV-2 structural glycoproteins towards ACE2 receptors governs its ability to infect cells more efficiently, thus spreading faster compared to other viruses [7]. Since SARS-CoV-2 particles use their spike glycoproteins to infect cells expressing ACE2 membrane receptors, the symptoms occur mostly in the lungs due to the high expression of ACE2. However, these receptors also exist on cells from other tissues, including arterial, intestinal, and heart tissues, which leads to the utterly complicated infection at the advanced stages of the disease [8]. On this basis, COVID-19 clinical manifestations could range from flu-like symptoms (i.e., fever and dry cough, myalgia, and dyspnea) to severe and systematic symptoms, leading to multiple organ failure and even death [9]. However, this virus can infect host cells and multiply less than the required threshold for the appearance of clinical symptoms (asymptomatic carrier). Therefore, the need for limited social contact became crucial, especially due to the virus’s long incubation period, reaching up to 14 days [10]. Accordingly, a precise detection tool was used for early detection in both symptomatic and asymptomatic patients. The nasopharyngeal swab test based on reverse transcription polymerase chain reaction (RT-PCR) of genes N1 and N2, encoding for nucleocapsid proteins, was the gold standard. Also, rapid tests based on immunological detection by using anti-nucleocapsid protein antibodies proved to be effective with nasopharyngeal samples [11].

The first line of defense was social distancing at the level of the individuals by keeping at least one meter away from one another. Similarly, quarantine and lockdown were established at the level of the community to avoid socially interactive activities [12]. Afterward, there was a rapid development of different types of vaccines targeting SARS-CoV-2 by sensitizing the immune system against it. In fact, vaccines could be made using attenuated whole viruses, recombinant viral protein fragments, or mRNA or DNA encoding for the viral antigens to be expressed directly on cells’ membranes [13]. Although the vaccines are generally safe, a series of side effects, including fever, pain in the injection site, and headaches, may prevail [14]. However, some vaccines are accompanied by rare but severe side effects. Specifically, after being vaccinated with Johnson Ad26.COV2 and AstraZeneca ChAdOx1 nCoV-19 vaccines, 39% of patients who suffered from vaccine-induced immune thrombotic thrombocytopenia (VITT) and cerebral venous sinus thrombosis (CVST) were deceased [15]. Also, other drugs have been suggested as a possible cure for COVID-19, including Remdesivir and Nirmatrelvir/Ritonavir, as they carry inhibitory effects against the virus. Unfortunately, these drugs can either cause adverse effects that vary among the different group categories or expose patients to the risk of having a drug-drug interaction leading to further complications [16,17].

**Figure 2:**
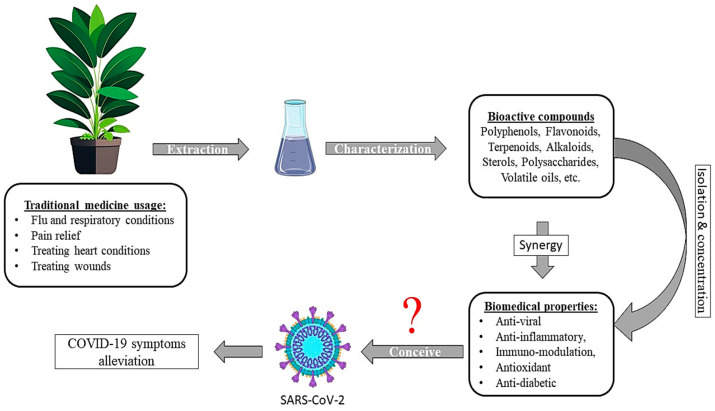
The potential of medicinal plants as a source of potential bioactive compounds against SARS-CoV-2 and COVID-19 symptoms management.

Our ancestors in Asia and Africa used to utilize various plants for medicinal applications to combat pathogens and infectious diseases [18]. Nowadays, therapeutic plants are under attention because of their low cost, fewer side effects, and good patient compliance [19]. Most importantly, plant-derived molecules can tackle viruses by acting on different aspects of their infection process. Various studies have identified herbs and natural compounds that can inhibit coronavirus/host protein pathways. These proteins include the spike S-protein, ACE-2, 3CL_pro_, nucleocapsid (N) protein, and cathepsin-L (CTSL). Drugs, herbs, or natural compounds able to inhibit or block one or more of these target proteins could interfere with the natural life cycle of SARS-CoV-2, providing antiviral properties [20]. The potential of medicinal plants could be assessed by extracting bioactive compounds and evaluating its effect against SARS-CoV-2, either individually or synergistically as shown in Figure 2. In fact, 45 plants proved to possess potent anti-viral activity against influenza and dermatology-related viruses (e.g., varicella, monkeypox, and herpes simplex viruses) [21]. Similarly, after a screening of 25 extracts taken from 8 different plants, *Ancistrocladus heyneanus*, *Bacopa monnieri*, *Plumeria alba*, and *Cucurbita maxima* showed anti-viral activity against chikungunya and dengue viruses [22]. Specifically, natural bioactive compounds such as terpenoids, flavonoids, phenols, alkaloids, etc. proved their high anti-viral potential against SARS-CoV-2 particles. Essentially, these biomolecules are capable of inhibiting the synthesis of non-structural proteins that are crucial for viral survival and multiplication in host cells by inhibiting 3CL_pro_ enzymes [23]. Additionally, plants’ extracts can inhibit this virus using a multitude of strategies, including the disruption of the viral adsorption capacity, impairing its entry to host cells, and preventing its replication, thus stopping its life cycle [24]. SARS-CoV-2 infection is linked to inflammatory disorders and the development of oxidative stress in extreme cases. Therefore, the anti-inflammatory and antioxidant properties that natural plants possess may alleviate these complications [19]. Flavonoids (e.g., quercetin, catechins, and their derivatives) are known for their anti-oxidant and anti-viral activity. These molecules are mostly effective against enveloped viruses and can inhibit SARS-CoV-2 by inhibiting 3CL_pro_ enzymes. In addition, essential oil phytocompounds containing low molecular weight and volatile molecules (i.e., monoterpenes, sesquiterpenes, alcohols, and ketones) proved their efficacy in disturbing the viral envelope due to their lipophilic properties, thus inhibiting their attachment to the host cell membrane [25,26]. Moreover, herbal infusions using sage and perilla showed a potent anti-viral effect via the activation of the heme oxygenase 1 enzyme (HMOX-1), leading to a reduction in oxidative stress, thus an inhibition of SARS-CoV-2 replication [27]. In addition, medicinal herbs are widely used to boost immunity against COVID-19, especially at an early stage of the disease, due to the presence of bioactive molecules, including vitamins and essential minerals. Equally, it can be used as a preventative due to its availability, low cost, and minimalistic side effects compared to synthetic drugs, which are not globally approved [28].

## 2. *Ginkgo biloba* (*G. biloba*)

*G. biloba* is a medicinal plant widely known for its phytotherapeutic and pharmacological properties. In fact, *G. biloba* extracts (GBE) govern various bioactive components collected from its dried green leaves [19,29]. Specifically, its extracts include flavonoids (quercetin, kaempferol, and isorhamnetin), biflavones (sciadopitysin and ginkgetin), terpene trilactones (ginkgolides and bilobalide), and alkylphenols (ginkgolic acids [GAs]). These molecules are responsible for GBE’s anti-inflammatory, antioxidant, and antiviral properties [30,31]. Thus, its antiviral activity could take place by affecting the life cycle stages of viruses in many different ways, including viral binding, fusion, viral entry, protein expression, and viral protein assembly and release. Moreover, GBE bioactive molecules, specifically GA, can deactivate viral particles by altering their genome and proteome [31]. Note that the plant displays a potent anti-influenza effect even below the cytotoxic threshold [32].

### 2.1. Inhibition of Viral Replication

The antiviral effect of GBE against SARS-CoV-2 is mediated by various mechanisms. First, its GA and bioflavonoids can block SARS-CoV-2 3-chymotrypsin-like protease (3CL_pro_), as observed in inhibition kinetic studies and docking simulations [33]. In addition, GBE effectively inhibited SARS-CoV-2 3CL_pro_ activity in in vitro experiments [34]. Since SARS-CoV-2 replication is mainly processed through the 3CL_pro_ enzyme, it constitutes a reasonable target that potentially blocks viral replication [35]. A molecular docking study by Cherrak et al. (2020) showed that glycosylated flavonoids present in GBE (i.e., quercetin and rutin derivatives) applied inhibitory effects on the replication of SARS-CoV-2 by forming inactive protein complexes with 3CL_pro_, thus constraining its enzymatic activity. Among the tested flavonoid derivatives, quercetin-3-O-rhamnoside showed the highest binding affinity for 3CL_pro_. However, regarding the dynamic behavior and stability of the protein in complex, rutin presented the highest potential to inhibit the function of 3CL_pro_ [36]. Second, papain-like protease (PL_pro_) is the second viral cysteine protease critical for the translation of the viral RNA, and therefore it is considered another potential antiviral drug target. Chen et al. (2020) found in an enzymatic inhibition assay that nontoxic concentrations of GA act as an irreversible inhibitor of PL_pro_ [37]. Likewise, quercetin, the main component of the GBE flavonoid fraction, is found to interfere with the replication of SARS-CoV-2 through inhibition of both 3CL_pro_ and PL_pro_ [38]. Third, kaempferol and quercetin, with their channel-blocking activity, inhibit SARS-CoV-2’s envelope protein E, consequently suppressing virus activity and proliferation [39].

### 2.2. Hypotensive Effect

*G. biloba* carries well-known hypotensive properties, specifically when SARS-CoV-2 has infected the host. Factually, ACE2 is involved in the control and downregulation of the renin–angiotensin system (RAS) by converting the vasoconstrictor and proinflammatory Ang II into Ang 1–7 and Ang 1–9. However, Ang 1–7 and Ang 1–9 are vasodilators and anti-inflammatories [40]. Consequently, the downregulation of ACE2 and the progression of high circulating Ang II during SARS-CoV-2 infection are the origins of inflammatory disorders and potential deleterious effects such as acute lung injuries (ALI) [41,42]. Interestingly, it is reported that GBE50 (44.1% of *G. biloba* total flavonoids and 6.4% of ginkgolides) extracted from leaves blocked the activation of local RAS through the moderation of overstimulated NF-κB/TLR4 pathway, thus lowering the increased expression levels of angiotensinogen and angiotensin type 1a (AT1a) receptors in cardiomyocytes [43]. In addition, some peptides isolated from GBE showed a potent inhibitory effect on ACE activity. ACE is the enzyme that converts Ang I to Ang II [44].

### 2.3. Anti-Inflammatory Effect

In SARS-CoV-2 infections, the dysregulation of inflammatory responses is marked by the activation of the node-like receptor pyrin 3 (NLRP3) inflammasome and T-helper 17 (Th17), which elevate proinflammatory cytokines and promote neutrophil recruitment [45]. Quercetin and other GBE constituents block the activation of NLRP3 inflammasome and Th17, thus reducing hyperinflammation and hypercytokinemia states. This helps in making COVID-19 symptoms milder by lowering inflammatory responses [46].

Also, quercetin-rich extracts trigger various biological effects, including anti-inflammatory by inhibiting proinflammatory mediators such as phospholipase A2 (PLA_2_) and lipooxygenase (LOX), antioxidant, and antiplatelet activities [47]. Through the regulation of both prostaglandin/leukotriene and threonine/serine kinases pathways, quercetin exhibits remarkable anti-inflammatory properties [48]. The co-administration of both vitamin C (500 mg) and quercetin (250–500 mg) twice a day could constitute a potent combinational therapy for preventing and treating COVID-19. Also, the immunomodulatory effects could be triggered by the initiation of cytokine storms. Consequently, this synergy inhibits the release of interleukin-6 (IL-6) and other proinflammatory cytokines. Fortunately, quercetin has a low cytotoxicity (CC_50_ = 3.32 μM) with an EC_50_ of 83.4 μM [49].

Quercetin and kaempferol can block the protein kinase phosphorylation and the expression of cation-selective channels (3A channel) in cells hosting SARS-CoV-2 particles. Additionally, they inhibit the release of proinflammatory cytokines, and the overexpression of reactive free radicals which lessens oxidative stress levels. Hence, with all the biological properties they offer, quercetin and kaempferol comprise efficient biocompounds to fight off COVID-19 [19].

## 3. *Curcuma longa* (*C. longa*, *turmeric*)

This plant belongs to the Zingiberaceae family and has various uses, such as textile dyes, herbal medicine, and food products. Many biological properties of its chemical constituents were reported, including anti-diabetic, anti-tumor, anti-inflammatory, antioxidant, etc. [50].

Curcumin is a yellow bioactive compound produced by the *C. longa* species. It is sold as herbal supplements, cosmetic ingredients, food flavoring, and coloring. Its medicinal properties have been known for thousands of years [51]. Curcumin has a wide range of therapeutic effects that make it an excellent candidate for use as adjuvant therapy in the treatment of patients with COVID-19. Note that curcuminoids have been approved by the united states food and drug administration (FDA) as “generally recognized ss safe” (GRAS) and have good tolerability and safety profiles [52]. Curcumin exhibits anti-inflammatory, antioxidant, antibacterial, antiviral, antifungal, anti-thrombotic, and antiproliferative properties [53].

### 3.1. Anti-Inflammatory Activity

Curcumin can downregulate the expression of various proinflammatory cytokines and chemokines, most likely through the inactivation of nuclear factor kappa B (NF-κB), thus reducing lung inflammation [54,55]. Curcumin leads to an increase in anti-inflammatory cytokines, showing that curcumin exerts beneficial effects by partially restoring the proinflammatory/anti-inflammatory balance during COVID-19 infection [56]. A well-characterized inflammasome NLRP3 is activated in conditions of tissue damage, metabolic stress, reactive oxygen species (ROS) overload, inflammation, and infection. By regulating NF-κB signaling, curcumin effectively suppresses the NLRP3 inflammasome, whose critical role is emphasized by recent studies in the immunopathogenesis of severe COVID-19, especially in patients with an increased risk of the disease (e.g., diabetes and obesity). Inflammasome activation can form a severe ‘cytokine storm,’ which causes acute respiratory distress syndrome (ARDS) and, eventually, death [51]. Nuclear factor erythroid 2-related factor 2 (Nrf2) is a central transcription factor that regulates the antioxidant defense system and is considered a modifier for several inflammatory diseases. It has been reported that curcumin is a promising nuclear factor erythroid 2–related factor 2 (Nrf2) agonist [57,58]. Hence, curcumin may exert antiviral activity against SARS-CoV-2 by activating the Nrf2 pathway [59].

### 3.2. Anti-Coagulant Activity

Procoagulant and pro-thrombotic events are recurrent in patients with COVID-19 and can cause significant damage [51]. Pawar et al. (2021) noticed that when curcumin is combined with antivirals and other blood thinners, those medications display higher efficacy. Regardless of the co-administration of heparin, the ingestion of curcumin (525 mg) extracted from the plant’s rhizomes with piperine (2.5 mg) twice a day would help improve COVID-19 coagulopathy. On this basis, curcumin treatment could reduce long-term thromboembolic complications in COVID-19 patients regardless of heparin treatment. Also, the thrombin inhibition and the reduction of blood viscosity play a key role in lowering coagulopathy in COVID-19 patients [60]. The study stated the absence of any adverse effects related to oxygen toxicity and thrombocytopenia, thus its long term administration is safe [53]. The anti-inflammatory and anti-thrombotic properties of curcumin could reduce the symptomatic manifestations of COVID-19 patients and accelerate their recovery.

### 3.3. Anti-Viral Properties

Curcumin presents potent antiviral effects which have been shown by its significant binding affinity towards SARS-CoV-2 proteins [53]. Modelling studies showed that curcumin inhibits the S-protein-ACE2 interaction in two ways [51]. Curcumin can either bind directly to the receptor binding domain of viral S-proteins or secure ACE2 receptors of the host cell. The disruption of S-protein and ACE2 receptors interaction impairs the ability of the virus to adhere to the host cell [61,62]. Therefore, the S-proteins of SARS-CoV-2 are considered as a possible target for drug therapies, in order to hinder viral adhesion to host cells and disrupt viral-host interactions.

In this framework, docking analyses showed that curcumin and a few of its derivatives possess promising potential as S-proteins inhibitors. A curcumin derivative (bis-demethoxycurcumin) displayed the best binding affinity to S-proteins of both SARS-CoV and SARS-CoV-2 [62]. Several studies showed that curcumin possess an efficient and sustainable binding affinity to the receptor-binding domain (RBD) of S-proteins and ACE2 receptors, leading to the inhibition of the viral attachment to the host cell [63,64].

In addition, Zhang et al. (2020) reported that curcumin could increase soluble ACE2 levels, which neutralize viral S-proteins and avoid their binding to cellular ACE2 receptors, thus impairing the viral cell entry. This would enhance the RAS and protect the lungs from injury [65,66]. In another study, among five major bioactive compounds of *C. longa* extracts curcumin had the highest binding affinity to SARS-CoV-2 main protease [67]. Based on in-silico studies, cyclocurcumin and curcumin extracted from turmeric’s rhizomes could significantly inhibit SARS-CoV-2 main protease enzyme, thus they may be efficient against COVID-19 [50].

## 4. *Artemisia annua* (*A. annua*)

*Artemesia annua* is an annual herbaceous plant used as a dietary spice, herbal tea, and medicinal plant. It has been used in traditional medicine for many years for the treatment of malaria and fever, in the form of tea or pressed juice. It is described as having anti-hyperlipidemic, anti-plasmodial, anti-convulsant, anti-inflammatory, anti-microbial, anti-cholesterolemic, and antiviral properties. Several bioactive components have been identified in *A. annua*, such as sesquiterpenes like artemisinin, arteannuin B, and artemisinic acid, and phenolic compounds like quercetin and rutin. Artemisinin and its derivatives can be used in the treatment of various diseases, such as cancer and viral infections. The antimalarial efficacy of artemisinin is significantly improved when combined with other compounds from *A. annua*, such as terpenes, flavonoids, and phenolic acids [68]. *A. annua* contains a vital compound known as artemisinin, a sesquiterpene lactone that exhibits low toxicity and is the parent compound for semisynthetic derivatives chemically modified at the C-10 position to give artesunate, artemether, arteether, artenimol (dihydroartemisinin), and artelinic acid [69].

### 4.1. Anti-Malarial Activity

Artemisinins include a series of well-known antimalarials with immune-modulatory activities [70]. Since the use of antimalarial medications such as chloroquine (CQ) and its derivative hydroxychloroquine (HCQ) to treat SARS-CoV-2 in vitro, questions were raised about whether other antimalarial drugs could be efficient against SARS-CoV-2. Factually, organic extracts of *A. annua* proved being more potent in treating malaria, as these natural extracts are faster and less toxic compared to CQ and HCQ [69]. Furthermore, the potent anti-SARS-CoV activity displayed by *A. annua* extracts suggests that they may also be active against SARS-CoV-2 [71].

### 4.2. Anti-Viral Activity

A recent controlled clinical trial investigated the anti-SARS-CoV-2 effects of artemisinin-piperaquine (AP). Patients diagnosed with COVID-19 were split into two groups, one of which received AP while the control received a combination of HCQ-arbidol. The AP group took significantly less time to reach undetectable levels of SARS-CoV-2 than the controls. Therefore, artemisinin-combination-based therapies (ACTs) could be a viable antiviral resource to aid in the treatment of SARS-CoV-2 infection [20].

Artemisinins are known for their extended-spectrum antiviral activity. For example, artesunate, an artemisinin derivative, is characterized by its antiviral efficiency against both DNA (e.g., human cytomegalovirus (HCMV), hepatitis B virus (HBV), and RNA viruses (hepatitis C virus (HCV) and human immunodeficiency virus (HIV)) [70,72]. Based on docking analyses and among various compounds such as rutin, artemisinin interacts with the 3CL_pro_ active binding sites with a good binding energy [73]. Another docking analysis showed that artesunate could also bind SARS-CoV-2 3CL_pro_ and remain stable with a significant binding energy compared to the Michael acceptor N3 inhibitor. This semi-synthetic compound was able to interact with five active sites of the enzyme, which might explain its remarkable binding affinity. Similar to SARS-CoV-2, Middle East respiratory syndrome coronavirus (MERS-CoV) CL_pro_ activity was efficiently inhibited by flavonoids, quercetin and di-caffeoylquinic acid which were extracted from *Artemesia annua* [73,74,75].

In addition, the two trimeric S-proteins of SARS-CoV-2 can firmly bind to the ACE2 dimers, specifically on Lys353 and Lys31 which are considered as important hotspots to the S-protein binding for viral entry [69]. In a recent in silico study, SARS-CoV-2 S-proteins were blocked by artemisinin derivatives. Particularly, artemisinin, artesunate, and artenimol could be considered as remarkable S-proteins inhibitor candidates, since they prevented these proteins from binding to Lys353 and Lys31 hotspots of ACE2. Therefore, these natural bioactive compounds could prevent the viral entry of SARS-CoV-2 into the host cell. After thorough analysis, the prioritization of artenimol for further clinical trials was recommended by the authors as in the body, most artemisinin derivatives are converted to artenimol [69,71]. Recently, a study on molecular dynamics using computer-aided drug discovery (CADD) showed that artemisinin and its derivatives showed more potency than HCQ. The same study revealed that the binding of artemisinin to the ACE2 hotspots remained stable and efficient against SARS-CoV-2 S-proteins [75].

Cathepsin L (CTSL), an endosomal protease, plays a key role in the membrane fusion and the internalization of SARS-CoV-2. On this basis, CTSL paves the path and opens new strategies for targeted therapies against this virus as it constitutes a potential therapeutic target. Interestingly, aurantiamide acetate (MOL736) is a compound extracted from *A. annua* that proved being an efficient inhibitor of the host CTSL protein [20]. N-proteins are essential for the virus as they help integrate its genomic material into the virions and proceed with its replication and transcription process. Therefore, targeting N-proteins may be another potential option for inhibiting the viral infection [70]. An immunofluorescence assay (IFA) performed on SARS-CoV-2 nucleoprotein showed that increasing concentrations of artemisinin inhibited the fluorescence of N-proteins in a dose-dependent manner compared to control. Precisely, *N*-proteins were completely inhibited when 25 μM of arteannuin B were added [70].

Artemisinin, extracted from the leaves, its semi-synthetic derivative artesunate (EC_50_ = 12.98 ± 5.30 μM), and its active metabolite dihydroartemisinin (EC_50_ = 13.31 ± 1.24 μM) have been shown to have antiviral potential. In fact, Artesunate is able to hinder viral infection by modifying the host cell’s metabolic pathways. Particularly, the efficacy of artesunate against HCMV is associated with the PI3-K/Akt/p70S6K signaling pathway. Also, artesunate is able to interact with cellular DNA-binding factors (e.g., NF-κB or Sp1) directly or indirectly, in order to inhibit viral replication. However, the same study assessed the cytotoxicity of these compounds and showed that artemisic acid, artemisinin, and artemisone have a CC_50_ of 200 μM [70].

### 4.3. Anti-Inflammatory and Pro-Immunogenic Properties

In vivo studies showed that artemisinins extracted from *A. annua* can reduce inflammatory cytokines levels including IL-6 and tumor necrosis factor alpha (TNF-α). The latter can cause complications in COVID-19 patients during the cytokine storm. Artemisinin can also reduce fibrosis, which is problematic and damaging to the patients’ organs. Arteannuin B showed the highest anti-SARS-CoV-2 effect displaying an EC_50_ = 10.28 ± 1.12 μM. However, arteannuin B is toxic at low concentrations with an IC_50_ of 10.3 μM [71]. In addition, besides blocking the viral entry into the cells, both arteannuin B and lumefantrine might disrupt intracellular signaling pathways that are yet to be identified [20].

*A. annua* stimulates adaptive immunity. Factually, it possesses the ability to stimulate CD8 and CD4 lymphocytes generation and increase CD4/CD8 ratio, in order to produce antibodies targeting SARS-CoV-2 antigens. Also, this leads to a downregulation in the production of proinflammatory cytokines prostaglandin E2 (PGE2), TNF-α, IL-6, and interleukin-10 (IL-10). Consequently, reducing cytokine storms could prevent the decrease in the number of Treg cells in COVID-19 patients and avoid the exhaustion of CD8 and CD4 lymphocytes, which leads to a more efficient and responsive immune system to tackle SARS-CoV-2. Furthermore, due to its high content of zinc, *A. annua* is effective for improving the immune response effectiveness by increasing CD4 levels. It should be noted that the antioxidant ability of Artemisia enhances immune defense [75].

## 5. *Nigella sativa* (*N. sativa*)

*N. sativa* has been used historically to treat various conditions including asthma, the common cold, headaches, nasal congestion, etc. Recently, black cumin seeds have been used to treat utterly complex conditions such as infections, cancer, diabetes, hypertension, obesity and gastrointestinal problems. In addition, *N. sativa* possesses antiviral, antioxidant, and anti-inflammatory activities [76]. The diversity of bioactive compounds that *N. sativa* presents is remarkable as it contains flavonoids, phytosterols, tannins, coumarins, and many other phenolic compounds. Specifically, this plant is rich in terpenes (e.g., thymoquinone (TQ), dithymoquinone (DTQ), carvone, limonene, t-anethole) and alkaloids (e.g., nigellidine, nigellicine, nigellicimine, nigellicimine-N-oxide, α-hederin) [77].

### 5.1. Anti-Oxidant Activity

The excessive production of ROS and the depletion of antioxidant systems are linked to the pathology of SARS-CoV-2 infection, which might cause multiple organ failure [76,78]. *N. sativa* has demonstrated some possible antioxidant properties that may help reduce oxidative damage in different organs. A clinical trial found that *N. sativa* oil significantly increased the levels of superoxide dismutase (SOD), which is a key antioxidant enzyme acting against oxidative stress in the body [79]. In addition, the bioactive components of *N. sativa* seeds like thymoquinone (IC_50_ = 211 μg/mL), carvacrol (IC_50_ = 28.8 μg/mL), and quercetin (IC_50_ = 1.31 μg/mL) showed variable antioxidant potency [80]. Note that thymoquinone is accompanied by many adverse effects, including a disruption of embryonic development in rats and an increase in chromosomal aberrations in the liver and kidneys. The LD_50_ for acute intoxication can range from 250 to 794 mg/kg in rats [81].

### 5.2. Anti-Inflammatory and Immune-Modulating Activity

*N. sativa* offers a variety of valuable properties such as antiviral properties, immune response modulation, enhancement in eosinophil counts and immunoglobulin E (IgE) serum levels, and the reduction of numerous pro-inflammatory cytokines (interleukin-4 [IL-4], interleukin-1b [IL-1b], IL-6, transforming growth factor beta [TGF-b], and interleukin-17 [IL-17]) [82]. In a randomized control trial, women suffering from obesity were treated with 3 g/day of *N. sativa* oil with calorie-restricted diet and displayed lower inflammatory biomarkers levels (TNF-α and C-reactive protein (CRP)) compared to control [83]. Similarly, elevated levels of systemic inflammatory cytokines and chemokines are observed in patients with severe COVID-19 [76]. Factually, the major cause of death among COVID-19 patients is the appearance of ARDS along with the rush of proinflammatory cytokines. *N. sativa* could be useful for the treatment of COVID-19 due to its anti-inflammatory, immunomodulatory as well as its protective effects on obstructive lung diseases [82]. Previous studies have also explained the anti-inflammatory activity of *N. sativa* through the inhibition of NF-κB which could alleviate the symptomatic outcomes of cytokine storms [76]. The TQ ingredient in *N. sativa* seeds has been shown to be the most potent anti-inflammatory. In fact, TQ was able to suppress prostaglandins and leukotrienes as inflammatory mediators. In addition, intraperitoneal administration of black cumin seed essential oil at 100 μL/kg inhibited 54% of edema in rat models [84].

### 5.3. Anti-Viral Properties

Many proteins and enzymes are showed to be good ligands for a-hederin extracted from the black cumin seeds. These proteins include: M_pro_, helicase [85], RNA-binding proteins [86], and endoribonuclease [87]. Surprisingly, the ability to bind to all these molecules would make this bioactive compound a very potent candidate to inhibit the viral life cycle through different mechanisms that end up disrupting its replication process [88].

The same study highlighted the binding efficacy of rutin, which was best-suited for the Nsp12 protein. This enzyme is an RNA-dependent RNA polymerase vital for replication and transcription, which rutin is likely to hinder. Similarly, Nigellamine A2 was found to be the best-suited ligand for Nsp3-papain-like protease, thus it carries a potent inhibitory potential against this protein [88]. Also, carvacrol extracted from *N. sativa* can block ACE2 receptors, thus inhibiting the SARS-CoV-2 entry into the host cells of [89,90]. It has been reported that the heat shock protein A5 (HSPA5), which play a key role as a recognition site for the SARS-CoV-2 S-proteins, constitutes also a high to moderate affinity target for TQ [91]. In addition, its other derivative, DTQ, presents a high binding affinity targeting SARS-CoV-2/ACE2 complexes. Therefore, it could be another potential candidate capable of hindering viral-host interactions [92,93]. Finally, nigellidine displayed a high binding affinity to a wide range of SARS-CoV-2 proteins including N-terminus-protease, non-structural protein 2, S-protein, and nucleocapsids, etc. Inflammatory biomarkers and other proteins such as human interleukin-1 receptor (IL1R), tumor necrosis factor receptor 1 (TNFR1), and tumor necrosis factor receptor 2 (TNFR2) were also remarkable targets for nigellidine binding [94]. Therefore, *N. sativa* constitutes a great source of secondary metabolites that could tackle SARS-CoV-2 infection and reduce its symptomatic complications.

## 6. *Zingiber officinale* (*Z. officinale*)

*Z. officinale*, also known as ginger, is a plant belonging to the Zingiberaceae family that is commonly used as a fresh ingredient in cooking or as a herbal treatment in traditional medicine [95]. Ginger contains a variety of bioactive compounds, among which phenolic compounds and terpenoids are the most interesting ingredients. Terpenoids extracted from ginger contain zingiberene, bisabolene, and curcuminoids, while its phenolic compounds are mostly represented by gingerols, shogaols, and gingerol-derived compounds such as zingerone [96]. Ginger carries a multitude of properties including antioxidant, anti-cancer, anti-diabetic, anti-inflammatory, and anti-viral activities, which makes it a popular ingredient in herbal medicine [97].

### 6.1. Anti-Oxidant Activity

Due to the presence of phenolic and terpenoid compounds, ginger leaves and rhizomes exhibit potent antioxidant activity, which has been reported in various studies. These studies proved its radical scavenging activity and antioxidant activity based on ABTS and DPPH assays, respectively [98,99]. Specifically, shogaols extracted from the rhizomes have higher antioxidant properties (7308 ± 131 μmol TE/g) compared to gingerols (4712 ± 166 μmol TE/g). This was attributed to the carbon chain length and the presence of unsaturated ketones [100]. In animal models, oral administration of ginger was shown to dramatically decrease oxidative stress biomarkers, including superoxide dismutase (SOD) and glutathione peroxidase (GPx) enzymes. Consequently, ginger is considered a carrier of potential anti-diabetic and neuroprotective molecules [101]. Fortunately, another study showed that shogaol had no adverse clinical effects on mice at dosages of 10, 20, and 40 mg/kg BW [102]. Since COVID-19 is associated with an overproduction of ROS through high oxidative stress, ginger constitutes a potential tool for reducing the stress on the cells, therefore lowering further complications. The increase in oxidative stress could also be caused by high TNF-α levels when the cytokine storm occurs due to other comorbidities alongside the SARS-CoV-2 infection [103]. Since 500 mg of ginger powder supplementation resulted in a decrease in cytokines levels, including TNF-α, this plant constitutes a potential source of bioactive molecules that reduce a variety of COVID-19 symptoms through scavenging free radicals and lowering cytokine levels in the blood [104].

### 6.2. Anti-Viral Properties

A docking analysis study showed that 6-gingerol, 8-gingerol, and 10-gingerol are great inhibitors of the PL_pro_ enzyme, which is essential for SARS-CoV-2 replication. In fact, these compounds have a great affinity in the order of nanomolars to completely bind PL_pro_ enzymes [105]. In addition, the 3-(4,5-dimethylthiazol-2-yl)-2,5-diphenyl-2H-tetrazolium bromide (MTT) assay showed that ginger’s methanolic extracts had a half-maximal inhibition concentration of IC_50_ = 206.4 μg/mL against the SARS-CoV-2 virus and a CC_50_ = 308 µg/mL, which confirms the non-toxic effect of the considered dose [106]. Additionally, ginger’s bioactive compounds (i.e., geraniol, shogaol, zingiberene, and zingiberenol) can disrupt the S-protein-ACE2 complex formation, thus inhibiting the ability of the virus to infect the host cells [107]. Another molecular docking analysis for 44 secondary metabolites extracted from red ginger showed that 27 compounds had a higher affinity for PL_pro_, while none of the compounds had any lower affinity for 3CL_pro_ when compared to the reference level [108]. Conversely, zingiberenol and zingiberol proved to be potent inhibitors of the M_pro_ enzyme, which is responsible for the cleavage and processing of polypeptides produced after viral mRNA translation [109]. To clarify these contradictory findings, another study performed a complete characterization of the methanol extract from *Z. officinale*’s rhizomes and showed that a bioactive compound (24-methylcholesta-7-en-3β-on) had a lower binding energy to 3CL_pro_. Further characterization showed that this molecule exhibited 75% inhibition of the SARS-CoV-2 3CL_pro_ enzymes, which was approximately similar to positive controls [110]. This indicated the ability of ginger extracts to target viral replication by inhibiting PL_pro_ and 3CL_pro_ and impair the viral infection by disrupting the S-protein-ACE2 binding.

### 6.3. Anti-Inflammatory and Anti-Thrombotic Activity

Ginger’s hot water extracts, administered to rats orally, showed a reduction in PGE2 and thromboxane at low concentrations (50 μg/mL), while also reducing cholesterol levels in the blood at 10 times this dose [111]. Specifically, *Z. officinale*’s polysaccharide extract proved to be a potent anti-inflammatory through the structural analysis of two of its polysaccharidic polymers [112]. In another study, gingerol and curcumin showed a high potency of immunomodulation through regulating TNF-α, NF-κ, and interferons. In addition, it promotes anti-inflammatory cytokines and regulates inflammation by maintaining cytokine and interleukin homeostasis. Since these two compounds are present in ginger, *Z. officinale* contains molecules that could regulate inflammatory responses within the SARS-CoV-2 infection, leading to the attenuation of severe COVID-19 symptoms [113]. The NLRP3 inflammasome is highly activated within pulmonary infections, which triggers a severe cytokine storm. Some bioactive compounds extracted from ginger (e.g., gingerols and shogaols) can inhibit NLPR3 inflammasome, which leads to a drastic reduction in cytokine levels, thus preventing the cytokine storm and avoiding many of the consequent symptoms [114].

## 7. *Allium sativum* (*A. sativum*)

*Allium sativum (A. sativum),* also known as garlic, is a well-known plant with long, green, and flat leaves. Their bulbs are aromatic and often used in cooking all over the world. Garlic cloves have been used in ancient times to treat a multitude of diseases including symptoms like fever, inflammation, gastrointestinal problems, skin eczema, rheumatism, and respiratory conditions (i.e., bronchitis and tuberculosis). Besides the fact that some of the previous properties could be helpful against virus-induced diseases, garlic is popular for its pure antiviral effects targeting viral life cycles [115,116]. Clinically, the ingestion of 24 g of garlic per day for three days for patients with moderate to severe COVID-19 symptoms was responsible for a significant alleviation of most of their disease manifestations, including fever and headaches, within the second day of administration [117].

### 7.1. Anti-Inflammatory and Immuno-Modulatory Activities

Garlic has a high anti-inflammatory effect due to the presence of a wide variety of sulfated bioactive molecules. In the context of combating COVID-19, the importance of this property relies on the inhibition of the cytokine storm through different mechanisms. First, methanolic extracts of aged black garlic inhibit the production of PGE2 and cyclooxygenase 2 (COX-2) via macrophage TLR suppression, leading to relatively lower NF-κB levels [118]. Second, it could occur by inhibiting the recruitment of granulocytes into the epidermis of the infected site. Third, the inhibition could target interleukins and chemokines production and free radicals’ reduction, which helps reduce inflammatory responses. Undoubtedly, this would not impair the immune system against pathogens. Allicin would enhance immune cells’ activity and offer stronger immunity against invaders with fewer symptomatic manifestations [119]. In addition, proteins extracted from *A. sativum* showed increased activation of lymphocytes T with more pronounced CD8+ lymphocyte proliferation in animal models. Also, human trials showed that these lymphocytes become more responsive to pathogen-associated molecular patterns (PAMP) after aged garlic ingestion [120]. Therefore, garlic’s active bioactive compounds are a great tool for COVID-19 symptoms’ alleviation.

### 7.2. Anti-Viral Activity

Organo-sulfur compounds (OSG) present in garlic, including allicin, ajoene, and garlicin, are shown to have potent anti-viral properties. Surprisingly, this includes a wide variety of viral families, including Adenoviridae, Flaviviridae, Herpesviridae, Orthomyxoviridae, Poxvirus, and most importantly, Coronaviridae. The anti-viral strategies could include host entry blockage inhibiting core enzymes (i.e., RNA polymerase and reverse transcriptase), thus disrupting viral replication [121,122]. In 2022, a molecular docking in silico study was performed in order to screen different compounds extracted from garlic that could tackle the SARS-CoV-2 virus by targeting 3CL_pro_ molecules. Accordingly, five compounds (squalene, 1,4-dihydro-2,3-benzoxathiin 3-oxide, 1,2,3-propanetriyl ester, trans-13-octadecenoic acid, and methyl-11-hexadecenoate) were found as potential therapeutic molecules against this virus [123]. Another docking study showed that 19 compounds extracted from garlic had a high affinity for ACE2, which indicates the presence of potential molecules that could constitute therapeutic inhibitors and novel preventative tools against SARS-CoV-2 [124]. However, further analyses are needed to verify their efficacy, spot their effective dose, and identify their adequate administration method in the hope of getting the most and the best out of *A. sativum.*

## 8. *Cinnamomum verum* (*C. verum*)

*Cinnamomum verum (C. verum)*, also known as cinnamon, is often used as a spice in cooking and other products such as toothpaste. It has also been used in pharmaceutics and traditional medicine for decades. Like most of the plants mentioned in this review, cinnamon is also rich in bioactive compounds that confer the ability to cure many varieties of diseases. Specifically, its richness in volatile oils (e.g., cinnamaldehyde, cinnamic acid, cinnamate, cinnamyl acetate, copane, and camphor) and the presence of eugenols as its major active compounds is responsible for its numerous medicinal properties, including antidiabetic, anti-HIV, anti-anxiety, anti-microbial, and anticancer activities [125].

### 8.1. Anti-Viral Activity

Cinnamon presents potent anti-viral properties against many different particles. Factually, essential oils extracted from *C. verum* at maximum non-toxic concentrations displayed potent activity against the herpes simplex virus (HSV-1) with an IC_50_ of 0.008%. The same study states that cinnamon’s essential oils disrupt the adherence of the virus to the cell surface by masking some of its proteins [126]. Similarly, cinnamon’s methanolic extracts contain cinnamaldehyde, cinnamyl acetate, and eugenol, which proved their ability to bind to ACE2 proteins in docking in silico studies. This suggests that these molecules hold the potential to inhibit the SARS-CoV-2 virus from binding to the cell, thus reducing its cellular infection. In addition, it was shown that different species of cinnamon could hold molecules that block the viral main protein M_pro_ and other spike proteins, disrupting both the entry and the proliferation of the virus through a lack of major components for the viral cell cycle [127]. In a recent study carried out in 2022, the half-maximal anti-viral activity of cinnamon’s essential oil against SARS-CoV-2 was approximately 50 μg/mL [128]. Moreover, the chemical characterization of *C. verum*’s water and ethanolic extracts showed the presence of 27 and 23 compounds, respectively. These extracts were able to inhibit ACE2 activity and SARS-CoV-2 spike protein’s binding in a dose-dependent manner. This indicates again the potential of cinnamon bioactive molecules in inhibiting the adherence of the virus to the host and possibly the inhibition of its replication by disrupting the main protease enzyme [129].

### 8.2. Anti-Diabetic and Anti-Atherosclerotic Activity

Cinnamon intake was proven to lower glucose absorption in the intestines by inhibiting pancreatic enzymes (i.e., α-amylase and α-glucosidase) responsible for the availability of glucose by breaking down dimers and polymers. Cinnamon’s bioactive compounds stimulate glycogenesis and inhibit neoglucogenesis, which leads to lower glucose levels in the blood. Therefore, this will help reduce the risk of developing insulin resistance, thus avoiding diabetes mellitus type 2 (DM-2). Moreover, in vivo animal models showed a reduction of low-density lipids (LDL) and an increase in high-density lipids (HDL), which reduces the amount of bad cholesterol and helps avoid atherosclerosis, alongside higher insulin levels produced in a fasting state, which reduces the accumulation of sugar in the blood [130]. Undoubtedly, hyperglycemia and hypercholesterolemia are risk factors that increase the mortality rate by four times in patients with COVID-19 disease compared to normal patients. This could be explained by the low efficacy of the immune system due to the high blood glucose, which leads to less phagocytotic activity and less neutrophil recruitment to the infection site. This helps the virus adhere and infect the host more easily [131]. Similarly, hypercholesterolemia is one of the comorbidities that should be considered when treating COVID-19 patients. In fact, statin therapies are being established alongside other cholesterol-lowering medications to avoid further cardiovascular complications [132]. On this basis, cinnamon could be a potent plant that could tackle SARS-CoV-2 by avoiding these comorbidities.

### 8.3. Anti-Depressive Activity

Based on the WHO, depression is projected to constitute a major cause of disability in 2030, so the need to tackle its manifestation became crucial [133]. Previous in vivo rat mice models showed that the administration of cinnamon essential oils at 2 mg/kg for two weeks significantly improved depression and anxiety behavioral symptoms. After the chemical characterization of cinnamon essential oils, 46 compounds were found, among which trans-cinnamaldehyde was the most prominent and probably the compound behind this activity [134]. The percentage of depression in COVID-19 survivors is between 11 and 28% in two weeks, which constitutes a major symptom of post-COVID-19 syndrome [135]. Accordingly, cinnamon might also be a helpful tool to reduce these COVID-19-induced depressive symptoms.

## 9. *Rosmarinus officinale* (*R. officinale*)

*Rosmarinus officinale (R. officinale)*, known as rosemary, is a plant from the Lamiaceae family. It is widely used due to its aroma in cooking and traditional herbal medicine. This plant has received the attention of researchers for decades due to its medicinal properties, including antioxidant activity and anti-inflammatory and analgesic properties. Its chemical characterization showed that rosemary is rich in diterpenoids and triterpenoids, which constitute its volatile essential oil responsible for its aroma. It also has other phenolic compounds (e.g., apigenin, diosmin, and luteolin) and phenolic acids, specifically rosmarinic acid and caffeic acid [136]. Moreover, a previous study in 2013 reported that carnosic acid extracted from *R. officinale* governs potential anti-viral properties, especially against the human respiratory syncytial virus (RSV). After mechanistic assessments, carnosic acid efficiently inhibits viral genetic expression [137]. This indicates that rosemary might carry potential bioactive compounds against respiratory SARS viruses.

### 9.1. Anti-Oxidant Activity

Rosemary extracts are rich in bioactive compounds due to their polyphenols and essential oil content. Several studies have shown that these compounds are good free radical scavengers, especially against lipids and active hydroxyls, leading to their stabilization. The two major compounds responsible for most of this activity are carnosic acid and carnosol. Their ability to increase superoxide dismutase and glutathione peroxidase stability and function might be the reason behind the anti-oxidant activity of carnosic acid. In another study, the 2,2-diphenyl-1-picryl-hydrazyl-hydrate (DPPH) assay showed that rosemary had anti-oxidant activity with an IC_50_ = 40.6 ± 2.6 μg/mL. Similarly, this was attributed to the presence of triterpenoids, betulinic, ursolic, and carnosic acids and their derivatives [138]. This suggests that rosemary might be a potential candidate to fight off free radicals’ accumulation that occurs within the SARS-CoV-2 infection. Factually, the accumulation of high levels of angiotensin 2 leads to higher levels of NADPH oxidase, which is majorly responsible for oxidative stress. With SARS-CoV-2 infection, ACE2 activity is disrupted, thus inhibiting the conversion of angiotensin 2 to angiotensin 1 and 7, which are responsible for the reduction of oxidative stress, cell damage, and even hypertension [139,140].

### 9.2. Anti-Viral Activity

A molecular docking analysis was performed to assess the binding affinity of 30 compounds extracted from rosemary to the SARS-CoV-2 main protease in comparison to remdesivir and favipiravir as positive controls. Mostly, apigenin, diosmin, betulinic acid, luteolin, and carnosol showed potent binding affinity for M_pro_, which indicates the possibility of using rosemary as an inhibitor and blocker of the SARS-CoV-2 life cycle by disrupting its replication inside the host [141]. Moreover, another molecular docking in silico analysis performed on different plants, including *Rosmarinus officinalis*, *Thymbra spicata*, *Satureja thymbra*, and *Stachys lavandulifolia* plants, showed that rosmarinic acid and rosmanol, which are found mostly in rosemary, hold a high potential to inhibit M_pro_ and ACE2. This proves again the ability of rosemary extracts to either prevent the infection of the virus by inhibiting the S-protein-ACE2 complexes or block viral proliferation by inhibiting its main protease enzyme [142].

### 9.3. Anti-Inflammatory and Analgesic Properties

In vivo rat models were performed to validate the anti-inflammatory activity locally using carrageenin-induced paw edema models and systemically using ischemic liver reperfusion models. In fact, the intravenous administration of 25 mg/kg rosmarinic acid extracted from dried leaves in the former model showed reduced local inflammation by reducing over 60% of the edema in the rat’s paw. Similarly, systemic inflammation was significantly reduced, along with a clear reduction in the level of multi-organ-damage biomarkers, including lung inflammation. This was shown to be via the modulation of NF-κB and metalloproteinase-9 [143]. This shows the great potential of rosmarinic acid extracted from rosemary in the reduction of both local and systemic inflammation, which suggests its ability to reduce the cytokine storm caused by SARS-CoV-2 infection. Additionally, volatile compounds of rosemary extracted from its dried leaves (n-hexane fraction at concentrations of 100 μg/mL) were responsible for a significant reduction of PGE2, which is responsible for inflammation and pain sensation. Note that the considered concentration displayed no cytotoxicity since the IC_50_ is 260.46 μg/mL [144]. On this basis, rosemary essential oils and volatile compounds are anti-inflammatory and analgesic agents, which would help reduce and manage the symptoms of COVID-19 by lowering cytokines and pain sensation.

## 10. *Taraxacum officinale* (*T. officinale*)

*Taraxacum officinale*, also called dandelion, is a common plant that grows in Europe and Asia. It is used for consumption in salads and drinks due to its nutritious content of vitamins, minerals, and fibers [145]. Also, it was widely used for its medicinal properties in ancient times; thus, its name derives from the Greek words taraxos (disorder) and akos (remedy). Dandelions present a multitude of benefits to the human body, as they can have antioxidant, anti-obesity, antidiabetic, antibacterial, and antiviral properties. This is due to the presence of flavonoids (e.g., quercetin, luteolin-7-glucoside), phenolic compounds (e.g., caffeic acid, chicoric acid), polysaccharides (e.g., inulin), terpenoids (e.g., taraxinic acid, α-amyrin), and sterols (e.g., taraxasterol) [146].

### 10.1. Antioxidant Activity

Dandelion leaves and roots showed high antioxidant activity in vitro and in vivo. Factually, the assessment using the microsomes P450 system as a biomarker of the antioxidant activity between different parts of the plant showed that the most potent extract from this plant is the ethyl acetate and water extract from its flowers. This activity is due to the presence of phenols and flavonoids that constitute a reducing activity of 40% in comparison to ascorbic acid. In in vivo models, rats had a low oxidative stress profile after being treated with *T. officinale* extracts [147,148]. Similarly, dandelions grown in Bulgaria displayed high antioxidant activity, which was proportional to the high content of phenolic compounds in the plant’s extracts. The same study stated that dandelion’s leaves constitute a rich source of cichoric acid, which is known for its antioxidant and radical scavenging activities [149]. Factually, the high-performance liquid chromatography (HPLC) analysis of dandelion leaves showed that cichoric acid constitutes 3148 mg/100 g DW in 50% ethanolic extracts. Since this compound is abundant in this extract, the radical scavenging activity of EC_50_ = 3.5 mg/mL is attributed to the presence of cichoric acid [150]. SARS-CoV-2 pathogenesis is related to a possible crosstalk between the cytokine storm induced by the viral infection and the generation of free radicals, which can damage red blood cells, leading to organ hypoxia, and the increase in free heme and iron levels, which is harmful to the cells. However, the oxidative stress can be caused by the virus directly, as the virus would possess the ability to alter the Nrf2 pathway to its advantage. Hence, the antioxidant activity of *T. officinale* constitutes a potential herbal remedy to help reduce the symptoms of COVID-19 by reducing the cytokine storm indirectly or by lowering the oxidative stress, which is advantageous for viral survival in the host [151].

### 10.2. Antiviral Activity

Dandelions present great potential to fight off avian viruses. In fact, their water extract showed a beneficial effect against influenza virus infection and lowered the polymerase activity in human lung adenocarcinoma cells (A549) at a concentration of C = 0.625 mg/mL without any adverse effect on the cells’ viability of metabolism. The study mentioned that dandelions’ water extracts could be titrated according to the patient’s symptomatic manifestations [152]. Regarding SARS-CoV-2 infection, high-molecular-weight compounds extracted with water from dandelions showed efficacy against spike proteins D614 and its different mutants (D614G, N501Y). For instance, it stops the interaction between the S1 viral proteins and ACE2 receptors on human kidney cells (HEK293) and human lung cells (A549), thus inhibiting viral adherence and internalization, which are essential steps in the viral life cycle. In addition, the same study showed that the infection of the extracts could prevent SARS-CoV-2 spike pseudotyped lentiviruses and prevent the initiation of the cytokine storm by lowering the levels of IL-6 triggered by the viral infection [153]. The same authors reported again that the mutation that led to the omicron variant did not affect the efficacy of that same extract but made the virus more sensitive and more susceptible to being prevented [154]. A novel study carried out in 2023 showed that silver nanoparticles with alcoholic and water extracts from T. offinicale’s roots displayed significant antiviral effects against SARS-CoV-2 and inhibited its viral activity. This inhibition was correlated with the inhibition of AP2-associated protein kinase 1 (AAK1), which allows viral entry. The silver nanoparticles could damage the ACE2 receptors by binding to the cell’s membrane due to its positive charge, leading to the impaired ability of the virus to adsorb and infect the host [155].

## 11. *Origanum vulgare* (*O. vulagre*)

*Origanum vulgare* (*O. vulagre*), also known as oregano, is a common plant belonging to the family Lamiaceae. Since it is a Mediterranean species, it can be found in many different countries, including Italy, Greece, and Egypt. This plant offers various bioactive compounds, such as essential oils (carvacrol, thymol, linalool, and p-cymene), polyphenols, and triterpenoids. These compounds confer many biological properties that are beneficial to human health; thus, oregano has been used by traditional medicine practitioners to cure a variety of diseases. It was used to cure cramps, coughs, expectorants, and intestinal flatulence. Nowadays, it is more evident to study the general effects that led to these different applications of the plant. It is evident that oregano possesses antiviral, antioxidant, anti-inflammatory, antispasmodic, and neuroprotective activities [156]. Since the effects of herbal extracts are broader and implicate multiple strategies to tackle COVID-19, oregano could be the key to a possibly potent way to prevent SARS-CoV-2 infection by disrupting its viral life cycle.

### 11.1. Antioxidant Activity

A study carried out in 2014 to identify phenolic compounds in oregano and assess their antioxidant and antiviral activities showed that this plant contains 21 phenolic compounds, of which 6 are newly characterized and 5 are known. Based on the DPPH radical scavenging assay, these compounds displayed great antioxidant activities of SC_50_ ranging from 16.7 ± 1.1 to 221.8 ± 49.0 μM for the compounds 1, 2, 7–9, 12–15, 18, and 19. Surprisingly, a newly discovered compound (1) had a higher radical scavenging activity compared to ascorbic acid control, which might be due to the presence of 3,4-dihydroxyphenyl and gastrodin as parts of the compound [157]. However, the antioxidant activity is affected by many different factors, including from which organ of the plant the extract is taken and if the plant is fresh or dried. Consequently, the DPPH assay showed that shade-dried oregano displayed higher antioxidant activity (31.48–32.71%) when compared to oven-dried oregano (26.19–27.87%). In addition, dried oregano constitutes a better source of antiradicals than fresh oregano. This is due to the presence of essential oils, which are also proportionate to the antioxidant activity of the extract. The yield of essential oils is significantly higher when the oregano is dried compared to fresh oregano. These essential oils containing 22 bioactive compounds are possibly behind this potent antioxidant activity since they represent 96% of the *O. vulgare* total oils [158]. Considering this antioxidant effect, the presence of less oxidative stress leads to an immunomodulatory effect due to the avoidance of chronic inflammatory responses and the persistence of cytokine storms through NF-κB and mitogen activated protein kinase (MAPK) signaling pathway regulation. These essential oils are key factors in tackling free radicals and regulating immune responses in order to avoid SARS-CoV-2 infection [159].

### 11.2. Antiviral Activity

Oregano offers significant antiviral activity against a wide variety of viruses, including DNA and RNA viruses. These include HSV-1, rotavirus, human respiratory syncytial virus (HRSV), and HIV-1. In addition, water and ethanol extracts from *O. vulgare* showed remarkable efficacy against equine influenza virus (EIV), canine coronavirus (CCoV), RSV, avian infectious bronchitis coronavirus I, and H1N1 viruses [160]. Interestingly, many of these particles target the respiratory system, some of which are coronaviruses. A recent in silico study assessed the inhibitory effect of essential oils extracted from different oregano species, including *O. vulgare*, against ACE2 receptors and LOX enzyme. The computational evaluation of the inhibitory effect showed that this plant can inhibit up to 74.3% and 81.1% of ACE2 and LOX molecules. Molecular docking analyses were performed in order to gain some insight about the morphological aspect of the inhibitory mechanism. This indicates the efficacy of essential oils extracted from oregano, specifically carvacrol, which constitutes the major component, in the disruption of the infection ability of SARS-CoV-2 and its entry into the host by binding to ACE2 receptors of the host and by LOX inhibition, which leads to less production of leukotrienes, which are important in the initiation and permanence of the cytokine storm (IL-1β, IL-6, IL-12, and TNF-α) induced by inflammatory responses [161,162]. The cytokine storm is what leads to symptoms and complications within the infection, including the long-term COVID-19 syndrome. Lowering the severity of the cytokine storm can be established by carvacrol from *O. vulgare*, which might relieve the symptoms and avoid systemic complications of the disease.

Finally, in Table 1, we summarize and present all the bioactive compounds with therapeutic potential against SARS-CoV-2 and derived from medicinal plants reviewed in this paper. We also describe the mechanisms of action of these compounds documented to date against SARS-CoV-2.

## 12. Discussion

In this comprehensive review, we discussed some properties of 10 different plants that possibly hold significant potential against SARS-CoV-2 infection. While other properties might also contribute to their beneficial effect against COVID-19 symptom severity, we highlighted the main biological activities of these plants, which are closely related to disrupting the life cycle of SARS-CoV-2 particles. Interestingly, the considered plants had multiple factors in common, which include the presence of phenols, flavonoids, and other bioactive compounds that allow the detection of remarkable antioxidant activity. Docking in silico studies showed that the 10 plants have bioactive compounds that present a high affinity towards ACE2 receptors present on the host cell’s membrane. In addition, some compounds were effective against M_pro_ and PL_pro_, which are essential for the viability and multiplication of the virus inside the host, which offers an additional layer of direct inhibition of the viral life cycle. Notably, other effects (i.e., anti-inflammatory, immunomodulatory, antidiabetic, etc.) can help combat COVID-19 symptoms and avoid major complications. Note that many in silico molecular docking analyses that proved the existence of high-affinity binding are scrutinized and filtered by other studies. Surprisingly, a study found that among 31 thousand bioactive compounds, only two proved to be effectively interacting with SARS-CoV-2. This is considered a major limitation in these docking analysis studies; therefore, in vitro and in vivo studies remain relatively better regardless of many other emerging limitations through the process [163].

Although these plants can offer these properties, their assessment in human clinical trials is not well documented, and the available studies are considered methodologically weak [164]. Factually, the chemical modification performed on the pants differs from the traditional herbal medicine used in ancient times; thus, multiple guidelines and problems should be considered prior to clinical trials of herbal drugs [165,166]. The administration of any kind of combination between conventional drugs and herbal extracts requires studying the presence of any adverse effects of the bioactive compounds on the cells or on patients with comorbidities should be considered. It was documented that many herbal drugs can interact with prescribed synthetic drugs, which might lead to major clinical complications for patients [167,168]. In addition, current studies tend to understand the individual effect of a specific bioactive compound, ignoring the fact that synergetic effects are essential to producing the targeted effect for which the plant is being used in ancient traditional medicine. This kind of synergy and interaction is a key factor in the plant’s ability to target a specific disease based on various mechanisms, which gives plants a significant advantage against synthetic drugs [169]. Furthermore, the extraction method and the solvent used for extraction should also be taken into consideration due to their impact on the nature of the bioactive compounds being extracted from the plant [170].

In conclusion, since these 10 herbs hold distinct bioactive compounds with significant properties in vitro and with remarkable benefits to human health, it is possible to prevent SARS-CoV-2 infection and reduce its symptomatic manifestations by consuming any of these 10 plants according to the recommended dose. The diversity in bioactive molecules between the different plants exerts various effects through different mechanisms at once, which makes it more potent than conventional synthetic drugs. Nonetheless, more studies are needed to highlight the clinical efficacy of these extracts and spot their possible side effects on patients, especially those with comorbidities who take multiple conventional drugs.

## Figures and Tables

**Table 1:** Summary of the major bioactive compounds and the molecular mechanisms against SARS-CoV-2 carried by the plants considered in this review.

Plant	Bioactive Compounds	Mechanisms against SARS-CoV-2
*Ginkgo biloba*	- Flavonoids: quercetin, kaempferol, isorhamnetin - Biflavones: ciadopitysin, ginkgetin - Terpenes: ginkgolides, bilobalide - Alkylphenols: ginkgolic acids (GAs)	- GAs block SARS-CoV-2 M_pro_ [36]. - GAs act as irreversible inhibitors PL_pro_ [37]. - Kaempferol and Quercetin inhibit SARS-CoV-2 envelope protein E [39].
*Curcuma longa*	- Curcumin	- Reduces proinflammatory cytokines, modulates NLRP3 inflammasome, and activates Nrf2 pathway [51,57]. - Inhibits thrombin and reduces blood viscosity [60]. - Inhibits viral binding to ACE2 receptors and by inhibiting Spike proteins [63,64]. - Inhibition of M_pro_ [50].
*Artemisia annua*	- Artemisinin, arteannuin B, artemisinic acid (sesquiterpenes) - Quercetin, rutin (phenolic compounds)	- Blockage of S protein receptor-binding domain [69]. - Inhibit SARS-CoV-2 3CL_pro_ [73]. - Arteannuin B inhibits the production of nucleocapsid proteins [70]. - Increase in CD4/CD8 ratio and reduction of the cytokine storm [75].
*Nigella sativa*	- Terpenes: thymoquinone (TQ), dithymoquinone (DTQ), carvone, limonene, t-anethole - Alkaloids: nigellidine, nigellicimine, nigellicimine-N-oxide, α-hederin	- Inhibition cytokine storms by reducing NF-κB [76]. - TQ suppresses prostaglandins and leukotrienes [83]. - Inhibition of Nsp3-papain-like protease, ACE2 and heat Shock Protein A5 [84,87,90].
*Zingiber officinale*	- Phenolic compounds (gingerols, shogaols, zingerone) - Terpenoids (zingiberene, bisabolene, curcuminoids)	- Decrease in oxidative stress biomarkers (SOD, GPx enzymes) [100]. - Reduction in inflammation (prostaglandin E2, thromboxane [109]. - Reduction in cytokines storm [112]. - Reduction in cholesterol levels [96]. - Gingerol and curcumin modulate immune responses [111]. - Disrupts ACE2 receptors, M_pro_ and PL_pro_ [103,108].
*Allium sativum*	- Allicin, ajoene, and garlicin	- Reduction in PGE2 and COX-2, lowering NF-κB levels [116]. - Reduction in the cytokine storm [117]. - Enhancement T lymphocytes activity against PAMPs [118]. - Blockage ACE2 receptors [122].
*Cinnamomum verum*	- Volatile oils: cinnamaldehyde, cinnamic acid, cinnamate, cinnamyl acetate, copane, camphor - Major active compound: eugenol	- Inhibition of ACE2, M_pro_ and spike proteins [125]. - Reduction in LDL, increases HDL, and prevents atherosclerosis reducing the risk factors for severe COVID-19 [128]. - Potential alleviation of COVID-19-induced depressive symptoms [131].
*Rosmarinus officinale*	- Rich in diterpenoids, triterpenoids, phenolic compounds (apigenin, diosmin, luteolin), and phenolic acids (rosmarinic acid, caffeic acid)	- Carnosic acid and carnosol stabilize free radicals [135]. - Carnosic acid, apigenin, diosmin, betulinic acid, luteolin, and carnosol block M_pro_ [138]. - Reduction in prostaglandin E2 thus lowering inflammation and pain [141]. - Counteract oxidative stress linked to high angiotensin 2 levels [136,137]. - Inhibit ACE2 receptors [139].
*Taraxacum officinale*	- Flavonoids: quercetin, luteolin-7-glucoside - Phenolic compounds: caffeic and chicoric acid - Polysaccharides: inulin - Terpenoids: taraxinic acid, α-amyrin - Sterols: taraxasterol	- Alcoholic and water extracts with silver nanoparticles inhibit ACE2 receptors [152]. - Efficacy against Omicron variant’s spike proteins is documented [151]. - Caffeic acid and chicoric acid reduce free radicals [146,147]. - Flavonoids reduce the cytokine storm [148].
*Origanum vulgare*	- Essential oils: carvacrol, thymol, linalool, and p-cymene	- Preventing cytokine storms through NF-κB and MAPK signaling pathway regulation [156]. - Carvacrol inhibits ACE2 receptors and lipoxygenase enzyme (LOX) [158,159]. - Regulating the cytokine storm (IL-1β, IL-6, IL-12, and TNF-α) [158,159].
